# Development and validation of a prognostic nomogram to predict the recurrence of AFP-negative and DCP-positive hepatocellular carcinoma after curative resection

**DOI:** 10.3389/fonc.2024.1414083

**Published:** 2024-08-08

**Authors:** Junnan Li, Qi Wang, Yadong Yan, Lina Sun, Gongming Zhang, Guangming Li, Ronghua Jin

**Affiliations:** ^1^ Beijing Key Laboratory of Emerging Infectious Diseases, Institute of Infectious Diseases, Beijing Ditan Hospital, Capital Medical University, Beijing, China; ^2^ People’s Hospital of Donghai County, Lianyungang, China; ^3^ Beijing Institute of Hepatology, Beijing You 'an Hospital, Capital Medical University, Beijing, China; ^4^ Department of General Surgery, Beijing You 'an Hospital, Capital Medical University, Beijing, China

**Keywords:** hepatocellular carcinoma, AFP negative, DCP positive, resection, recurrence

## Abstract

**Purpose:**

Approximately one-third of hepatocellular carcinoma (HCC) cases are characterized by alpha-fetoprotein (AFP) negativity (AFP-NHCC. Among these patients, around 60% exhibit des-gamma-carboxyprothrombin (DCP) positivity, and DCP-positive patients have a poorer prognosis. As a curative treatment, recurrence after liver resection poses significant challenges to the prognosis of HCC patients. Therefore, it is necessary to determine the relevant risk factors of these patients and provide timely treatment options.

**Methods:**

This study included 540 patients who underwent resection at Beijing You’an Hospital. 292 patients from 2014 to 2018 constituted the training cohort, while 248 patients from 2018 to 2020 constituted the validation cohort. All patients underwent routine follow-ups until December 2023. Variables were identified through Cox regression, and a nomogram was developed. The nomogram was evaluated using time-dependent receiver operating characteristic curves (ROC), calibration curves, Decision curve analysis (DCA), and Kaplan-Meier (KM) curve analysis

**Results:**

We found that age, tumor number, tumor size, γ-glutamyl transpeptidase (γ-GT), and prothrombin time (PT) are independent risk factors for HCC recurrence, and a nomogram was developed and validated based on this result to predict recurrence-free survival (RFS) at 1, 2, and 3 years. The performance of the nomogram was further confirmed by the ROC curve, calibration curve, and DCA, all of which showed favorable results. The KM curve analysis clearly distinguishes between two groups of people with different risks in terms of prognosis in both the training and validation sets.

**Conclusion:**

In summary, we established and validated a novel nomogram by multivariate Cox regression analysis to predict recurrence in DCP-positive patients with AFP-NHCC after resection. The nomogram, including age, tumor number, tumor size, γ-GT, and PT, demonstrates better predictive ability for AFP-NHCC patients with DCP positive.

## Introduction

Hepatocellular carcinoma (HCC), the most common primary liver cancer, is one of the most malignant diseases in the world, with the sixth most common cancer worldwide and the third leading cause of cancer death in the world. There were approximately 906,000 new cases and over 830,000 deaths from HCC in 2020, with 410,000 new cases and 390,000 deaths. China accounts for half of these new cases and deaths, suggesting a universally poor prognosis (1, 2). In China, the main pathogenic factor is chronic HBV infection or aflatoxin exposure (3). For patients with early-stage HCC, there are a few curative therapy options currently available, including surgical resection, liver transplantation, and local ablations (4–6). Surgical resection is the primary treatment for HCC, but they are only effective in 15%-30% of cases (7, 8). The most significant factor affecting treatment outcomes is tumor recurrence after surgery. According to the research, the postoperative recurrence rate at 5 years is reported to be >70%, which seriously affects the survival and prognosis of patients (9).

Serum alpha-fetoprotein(AFP)is considered the most reliable biomarker of HCC diagnosis, it is widely applied for the screening, diagnosis, and monitoring of HCC recurrence and metastasis (10, 11). However, approximately one-third of HCC cases are characterized by AFP negativity (AFP-NHCC) (AFP < 20 ng/mL) (12), which poses challenges to the diagnosis and treatment of patients with AFP-NHCC and adversely affects their prognosis (13, 14). In these patients, a considerable proportion exhibit elevation of des-gamma-carboxyprothrombin (DCP) level (>40mAU/ml) (15, 16). DCP, also known as a protein induced by vitamin K absence or antagonist-II (PIVKA-II), is an aberrantly elevated prothrombin protein in the serum of HCC patients. Currently, in the Asia-Pacific region (such as Japan and South Korea), DCP has been listed as a specific indicator for diagnosing HCC (17–20). Moreover, research indicates that the prognosis of AFP-negative and DCP-positive patients is only slightly inferior to that of double-positive patients for AFP and DCP (21).

Some indicators are predicting the prognosis of AFP-NHCC patients, such as Barcelona Clinic Liver Cancer (BCLC) staging and American Joint Committee on Cancer (AJCC TNM) staging, TBS-PIVKA II score, ALBI, MLR, and NrLR (22–24), as their descriptions of diseases are not comprehensive enough due to the introduction of heterogeneity. Nomograms have emerged as a simpler and more advanced method. The main advantage of nomograms is their ability to assess individual risk based on patient and disease characteristics (25). Currently, many studies have utilized nomograms to predict recurrence-free survival (RFS) and overall survival (OS) in AFP-NHCC patients after surgical resection (26, 27). However, there is a lack of research in the field for AFP-NHCC patients with elevated DCP levels. Therefore, there is an urgent need to identify relevant risk factors and establish a comprehensive nomogram to predict recurrence in this specific subtype of patients. and offer timely treatment choices for this subset of patients.

## Patients and methods

### Patients

This study included 540 AFP-NHCC patients with elevated DCP levels who underwent liver resection at Beijing You’an Hospital, Capital Medical University. 292 patients from 2014 to 2018 constituted the training cohort, while 248 HCC patients from 2018 to 2020 constituted the validation cohort.

Inclusion criteria for this study were as follows (1): The pathological diagnosis was HCC (2); Child-Pugh stage A or B and BCLC stage 0 or A (3); Patients received radical hepatectomy (4); The serum AFP level < 20 ng/mL and DCP level > 40mAU/ml before hepatectomy. Exclusion criteria included as follows (1): Had received other treatments before surgery (2); Have distant metastasis or other malignant disease (3); Have other primary malignancies (4); Patients with Vit. K or warfarin administration (5); Before recurrence, have other treatment after surgery (6); Clinical follow-up data are incomplete

As a retrospective study, the study protocol was approved by the Ethics Committee of Beijing You’an Hospital and complied with the requirements of the Declaration of Helsinki. As a low-risk study by the Helsinki protocol, written informed consent was waived by the same local committee.

### Laboratory examination and clinicopathologic data

Clinicopathological data and baseline data were obtained from the electronic medical record system, including (1) demographics and medical history: age, gender, hypertension, diabetes, antiviral, smoking, drinking, and cirrhosis, (2) liver function and coagulation action test: total bilirubin (TBIL), direct bilirubin (DBIL), albumin (ALB), aspartate aminotransferase (AST), alanine aminotransferase (ALT), γ-glutamyl transferase (γ-GT), prothrombin time (PT), and Child-Pugh grade, (3) complete blood count: white blood cells (WBCs), red blood cells (RBCs), and hemoglobin (Hb). (4) tumor information: tumor number, tumor size, and BCLC stage.

### Follow-up

All patients were routinely followed up after liver resection until December 2023. After hepatectomy, abdominal CT or MRI, AFP levels, and other laboratory examinations should be performed. Follow-up was conducted every 3 months for the first two years and every 6 months thereafter. The endpoint of the study was recurrence-free survival (RFS), which was the time from hepatectomy to the first detection of recurrence, the time of death without detection of HCC recurrence, or the last observation time.

### Statistical analysis

Statistical analysis was done using SPSS 26.0 and R 4.1.2 software. Categorical variables are presented as numbers (percentage) and compared using chi-square, while continuous data are expressed as mean ± standard deviation (SD) and analyzed by Student’s t-test. Univariate and multivariate COX regression analyses were used to identify independent risk factors for recurrence. The recurrence curve was plotted by the Kaplan-Meier (KM) method, and the data were compared by log test. Nomogram was developed by R software. Discrimination and calibration of the nomogram were performed by the training cohort and verification cohort respectively. Decision curve analysis (DCA) was performed to determine the clinical decision benefit of the nomogram. A two-tailed P value < 0.05 was considered statistically significant.

## Results

### Basic characteristics

To develop and validate the nomogram, we assigned 292 patients from 2014 to 2018 to the training cohort and 248 patients from 2018 to 2020 to the validation cohort. The clinicopathological features of the training and validation cohorts were evaluated. **Table 1** shows the characteristics of 292 patients in the training cohort and 248 patients in the validation cohort. Furthermore, the absence of statistically significant differences in baseline variables between the two cohorts suggests a high level of consistency.

**Table 1:** Patient clinical characteristics in the training cohort and validation cohort.

Characteristics	Training Cohort (N = 292)	Validation cohort (N = 248)	*P* value
Age— no. (%)			0.991
≤60 years	180 (61.6)	153 (61.7)	
>60 years	112 (38.4)	95 (38.3)	
Gender— no. (%)			0.438
Male	243 (83.2)	200 (80.6)	
Female	49 (16.8)	48 (19.4)	
Hypertension— no. (%)	75 (25.7)	75 (30.2)	0.239
Diabetes— no. (%)	84 (28.8)	70 (28.2)	0.890
Etiology— no. (%)			
HBV/HCV	268 (91.8)	218 (87.9)	0.134
Other	24 (8.2)	30 (12.1)	
Antiviral— no. (%)	188 (64.4)	166 (66.9)	0.534
Smoking— no. (%)	129 (44.2)	110 (44.4)	0.967
Drinking— no. (%)	111 (38.0)	79 (31.9)	0.135
Cirrhosis— no. (%)	266 (91.1)	224 (90.3)	0.757
BCLC stages— no. (%)			0.420
0	109 (37.3)	101 (40.7)	
A	183 (62.7)	147 (59.3)	
Child-Pugh class— no. (%)			0.190
A	218 (74.7)	197 (79.4)	
B	74 (25.3)	51 (20.6)	
Tumor number — no. (%)			0.440
Single	240 (82.2)	210 (84.7)	
Multiple	52 (17.8)	38 (15.3)	
Tumor size — no. (%)			0.647
≤3 cm	202 (69.2)	167(67.3)	
>3 cm	90 (30.8)	81(23.7)	
WBC (mean ± SD),10^9^/L	5.02 ± 2.21	5.11 ± 2.13	0.625
RBC (mean ± SD),10^6^/L	4.15 ± 0.61	4.22 ± 0.6	0.181
Hb (mean ± SD),g/L	130.33 ± 19.51	131.24 ± 19.41	0.590
Total Protein (mean± SD), g/L	64.91 ± 8.33	65.96 ± 7.64	0.130
Globulin (mean ± SD), g/L,	27.72 ± 4.97	28.55 ± 5.08	0.056
γ-GT (mean ± SD), U/L	62.72 ± 47.73	61.82 ± 53.61	0.837
PT (mean ± SD), s	12.24 ± 1.48	12.37 ± 1.5	0.573

BCLC, Barcelona Clinic Liver Cancer; WBC, white blood cell; RBC, red blood cell; Hb, Hemoglobin; γ-GT, γ-glutamyltransferase; PT, prothrombin time.

### Independent prognostic factors for RFS

To investigate the risk factors associated with RFS, univariate analyses were performed on 27 clinical variables. The results showed that (**Table 2**), age, tumor number, tumor size, γ-GT, PT, Child-Pugh, BCLC, and Hb were significantly correlated with RFS. Through further Cox multivariate analysis, we found that age (HR:1.016; 95%CI: 1.001-1.032), tumor number (HR: 1.552; 95%CI: 1.016-2.371), tumor size (HR:1.638; 95%CI: 1.108-2.421), γ-GT (HR:1.006; 95%CI: 1.003-1.009), PT (HR: 1.174; 95%CI: 1.046-1.317) are independent risk factors for HCC recurrence.

**Table 2:** Univariate and multivariate Cox analysis of the training cohort.

Variables	Univariate analysis			Multivariate analysis		
	HR	95%CI	*P* value	HR	95%CI	P value
Age	1.015	1.000-1.030	**0.043**	1.016	1.001-1.032	**0.040**
Sex	0.773	0.534-1.121	0.175			
Hypertension	0.844	0.618-1.154	0.288			
Diabetes	0.876	0.617-1.245	0.461			
Etiology	1.025	0.996-1.055	0.086			
Antiviral	0.890	0.681-1.163	0.394			
Smoking	1.102	0.842-1.442	0.480			
Drinking	1.255	0.955-1.648	0.103			
Cirrhosis	1.107	0.780-1.572	0.570			
ChildPugh	1.336	0.989-1.805	**0.059**	0.912	0.641-1.297	0.607
BCLC	1.483	1.121-1.963	**0.006**	1.051	0.703-1.571	0.807
t.n.	1.363	0.966-1.923	**0.078**	1.552	1.016-2.371	**0.042**
t.s.	1.670	1.260-2.215	**<0.001**	1.638	1.108-2.421	**0.013**
WBC	0.981	0.922-1.043	0.535			
RBC	1.17	0.65-1.12	0.26			
Hb	0.767	0.632-0.945	**0.013**	1.001	0.993-1.009	0.848
PLT	0.998	0.996-1.000	0.129			
ALT	1.003	0.997-1.008	0.387			
AST	1.000	0.99-1.01	0.829			
AST/ALT	1.009	0.994-1.025	0.242			
TBIL	1.000	0.98-1.01	0.707			
DBIL	1.010	0.95-1.02	0.533			
Total Protein	0.991	0.976-1.007	0.255			
ALB	0.970	0.94-1.01	0.195			
Globulin	1.020	0.99-1.06	0.148			
γ-GT	1.006	1.003-1.008	**<0.001**	1.006	1.003-1.009	**<0.001**
PT	1.149	1.050-1.258	**0.003**	1.174	1.046-1.317	**0.007**
INR	0.62	0.5-5.11	0.425			

Bold represents statistical significance, with univariate Cox <0.1 and multivariate Cox <0.05.

### Prognostic nomogram for RFS and the evaluation of the nomogram

**Figure 1:**
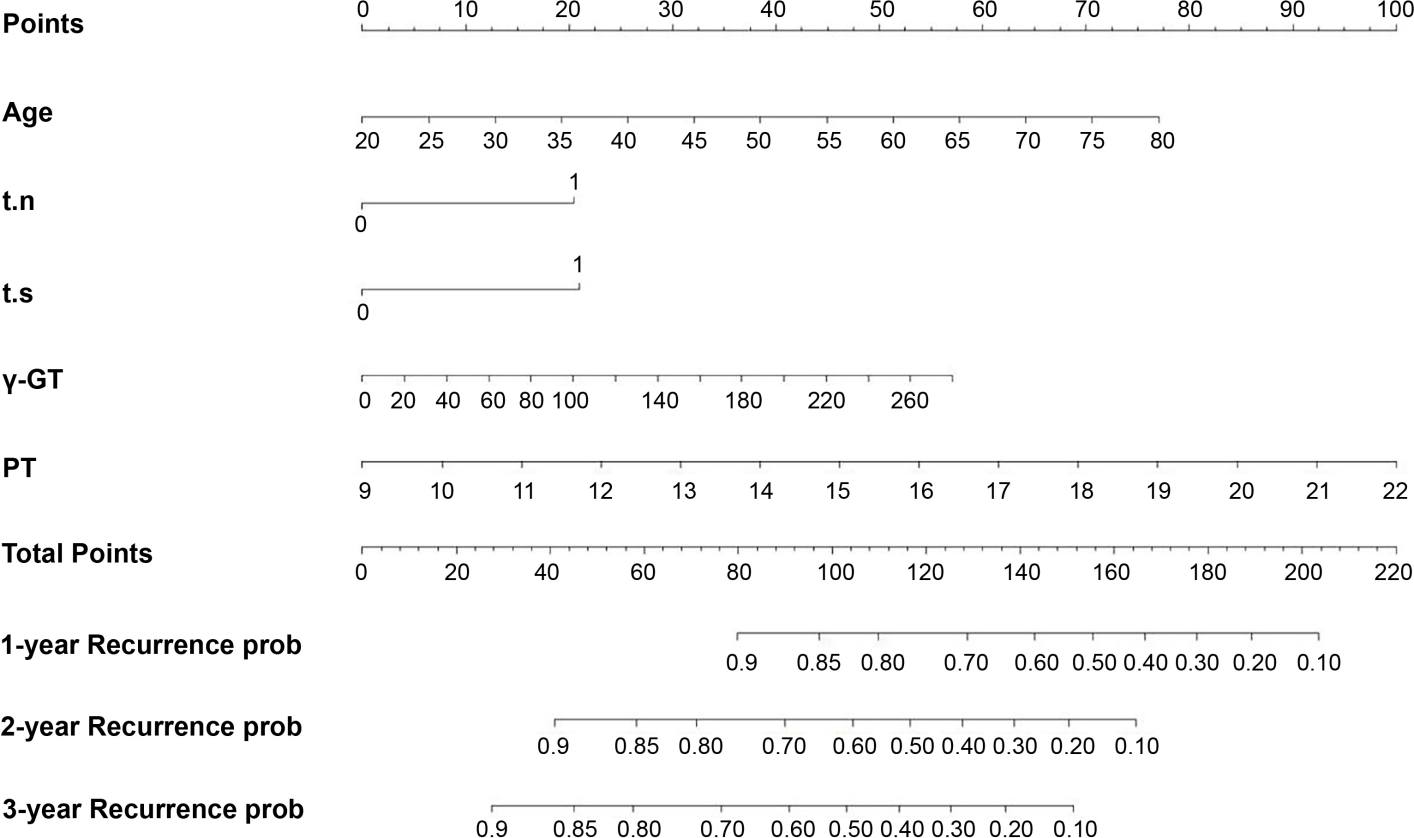
Nomogram, including Age, t.n, t.s, γ-GT, and PT, for one, two, and three years RFS in patients with AFP-NHCC and elevated DCP level. The nomogram is valued to obtain the probability of 1, 2, and 3 years’ recurrence by adding up the points identified on the points scale for each variable. t.n, tumor number; t.s, tumor size; γ-GT, γ-glutamyl transpeptidase; PT, prothrombin time.

Based on the five identified risk factors, a nomogram was constructed to predict RFS at 1, 2, and 3 years after resection (**Figure 1**). The nomogram is valued to obtain the probability of 1, 2, and 3 years of recurrence by adding up the points identified on the points scale for each variable.

**Figure 2:**
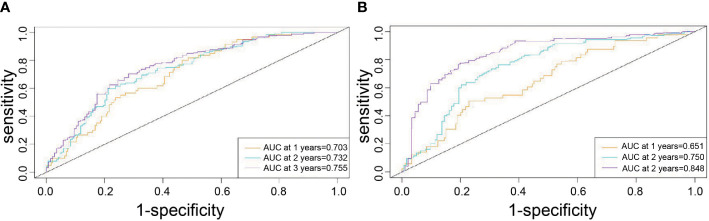
Time-point roc curves of the nomogram in the training and validation cohort. **(A)** The AUCs for RFS at 1, 2and 3 years in the training cohort. **(B)** The AUCs for RFS at 1, 2and 3 years in the validation cohort.

**Figure 3:**
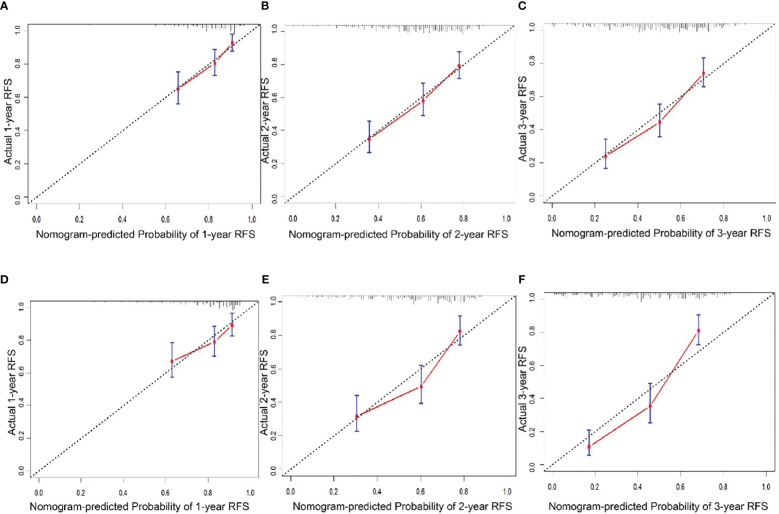
Calibration curve of the nomogram in the training and validation cohort. A.1-year RFS in the training cohort. **(B)** 2-year RFS in the training cohort. **(C)** 1-year RFS in the training cohort. **(D)** 1-year RFS in the validation cohort. **(E)** 2-year RFS in the validation cohort. **(F)** 3-year RFS in the validation cohort.

In the training cohort, the median follow-up time was 67.63 months (11.1-114.5) and the median RFS was 30.4 months (2.37-112.6). The Harrell’s concordance index (C-index) for RFS prediction was 0.785 (95%CI = 0.748-0.822). Time-point receiver operating characteristic (ROC) curves are used to evaluate the discrimination ability of the nomogram. The AUCs of ROC curves for RFS at 1, 2, and 3 years were 0.703,0.732, and 0.755(**Figure 2A**). In addition, the calibration curves for RFS at 1, 2, and 3 years confirmed a high degree of agreement between nomogram predictions and actual observations (**Figures 3A–C**).

### Analysis of the nomogram in the validation cohort

In the validation cohort, the median follow-up time was 34.8 months (4.6-54.7) and the median RFS was 23 months (1.4-54.7). The C-index for RFS prediction was 0.799 (95%CI = 0.75-0.848). Time-point roc curves are used to evaluate the discrimination ability of this nomogram. The AUCs (ROC curve) for RFS at 1, 2, and 3 years were 0.651,0.75 and 0.848 (**Figure 2B**). After hepatectomy, the calibration curves for RFS at 1, 2, and 3 years confirmed a high degree of agreement between nomogram predictions and actual observations (**Figures 3D–F**).

### Decision curve analysis

**Figure 4:**
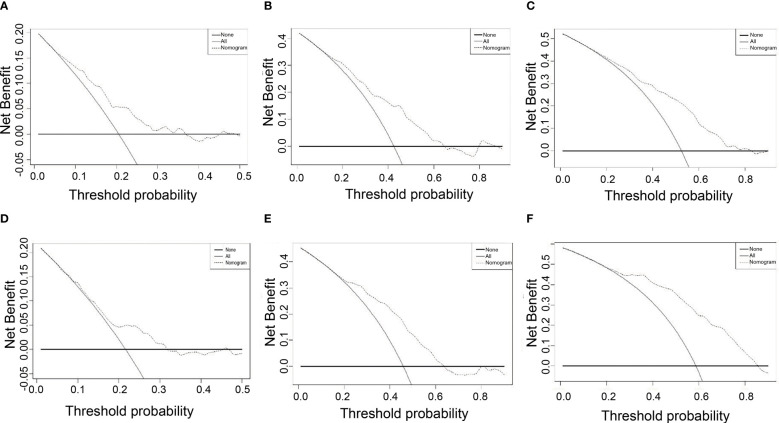
DCA for nomogram depicts the clinical net benefit. **(A)** 1-year DCA in the training cohort. **(B)** 2-year DCA in the training cohort. **(C)** 1-year DCA in the training cohort. **(D)** 1-year DCA in the validation cohort. **(E)** 2-year DCA in the validation cohort. **(F)** 3-year DCA in the validation cohort.

DCA displays the benefits obtained using a predictive model at different treatment decision thresholds. It plots the sensitivity and specificity of the predictive model on the x-axis against the net benefit on the y-axis. The net benefit of the reference strategy is set to zero, meaning its decision curve always lies below the x-axis. The DCA curve of this nomogram being further away from the decision curve of the reference strategy indicates the higher clinical utility. According to the DCA of this nomogram, if a patient’s threshold probability is closer to 10% or higher, this nomogram is more beneficial in predicting RFS whether all patients with recurrence or no patients with early recurrence (**Figure 4**).

### Risk strata of nomogram

**Figure 5:**
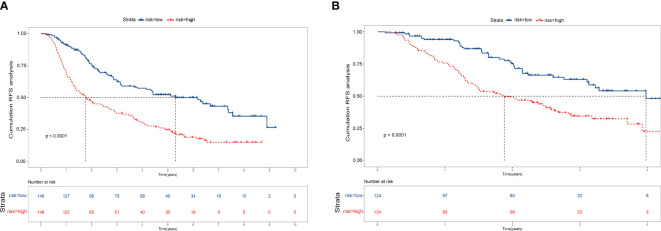
Kaplan–Meier survival curves for patients with high- and low-risk recurrence after resection by the nomogram score. **(A)** Training cohort. **(B)** Validation cohort.

Finally, we calculate the total risk score for all patients based on the nomogram. Then, using the median score, we divide the patients into low-risk and high-risk groups. Kaplan-Meier curves demonstrate distinct prognostic categorization for HCC patients in these two distinct risk groups. This stratification approach was similarly employed for patients in the validation cohort, with all results showing statistical significance (P < 0.001). (**Figure 5**)

## Discussion

HCC, as the primary type of liver cancer, is a leading cause of cancer-related deaths worldwide. this increases the length of hospital stays for patients and severely impacts their prognosis (28). AFP-NHCC with DCP level elevation exhibits a worse prognosis compared to other subgroups where these two indicators do not show elevation. Therefore, it is of paramount importance to identify high-risk patients prone to recurrence after hepatectomy and to initiate early monitoring along with appropriate clinical decisions.

In our study, we included 540 patients with AFP-NHCC with elevation of DCP level who underwent hepatectomy. We identified risk factors for early HCC recurrence and used these factors to establish a nomogram to predict the recurrence of HCC. This model based on multivariate Cox regression analysis shows effective prediction performance, as supported by high AUCs of the time-dependent ROC curves for the training and validation cohorts, especially for the 2- and 3-year time points. The calibration curves displayed good agreements, suggesting predictive accuracy for our nomogram, and the DCA curves confirmed a high net clinical benefit rate. In addition, the clinical indicators applied in this study cover a wide range, including demographics, liver function, and tumor load, allowing for a more comprehensive assessment of patients. What’s more, our nomogram has high guiding value to the clinic, the model is not only simple and easy to obtain but also helps clinicians to assess patients and treat them precisely in the shortest time, such as considering repeat surgical resection, liver transplantation, ablation, and close follow-up.

In univariate Cox regression, it showed a significant correlation between Child-Pugh score, BCLC stage, HB, and RFS. Child-Pugh stage as grading of liver reserve function in cirrhotic patients has significant roles in prognostic prediction for HCC patients and is associated with an increased risk of unfavorable outcomes. BCLC stage is the most widely used staging system worldwide and has been validated in several large studies for prognostic prediction in patients with HCC, especially early HCC (29, 30). However, these variants were not significant in multivariate Cox regression. This nomogram contains five independent risk factors for early recurrence, namely age, tumor number, tumor size, γ-GT, and PT. Several possible mechanisms can explain the rationale of our model. Advanced age has been confirmed by numerous studies as an independent risk factor for adverse postoperative outcomes in many diseases (31). This is mainly due to the lower immune response and physical resilience in elderly patients, leading to slower postoperative recovery and an increased risk of recurrence. One study showed that tumor size >5 cm may be associated with higher recurrence and lower survival (32). Multiple tumors are more prone to microvascular infiltration (MVI) than single tumors, which may lead to an increase in postoperative tumor recurrence. Additionally, GGT is a glycosylated mitochondrial transferase located on the cytoplasmic membrane of liver cells (33). When the body undergoes oxidative stress, GGT levels significantly increase. However, high levels of GGT can, in turn, affect the glutathione (GSH) antioxidant balance, thus promoting the development and progression of tumors (34, 35). Serum GGT levels were demonstrated to increase with hepatocarcinogenesis and promote tumor progression in an animal HCC model (36). In addition, it has been established that GGT is an independent risk factor for predicting postoperative recurrence of liver cancer and has value in the early diagnosis of HCC (37–39). And, another study (40) showed that GGT has no correlation with AFP concentration or tumor size, and in AFP-NHCC patients, the positivity rate reaches 75%, which can compensate for the deficiency of AFP. Therefore, GGT not only serves as a complement to the diagnosis of AFP-NHCC but also has a certain impact on the prognosis of AFP-NHCC patients. The liver plays a crucial role in the metabolism and synthesis of clotting factors, and when the liver is damaged, the synthesis of both clotting factors and anticoagulant proteins can be impaired (41). HCC patients experience disease progression, cancer cell proliferation, and infiltration, decreased immune function, and hindered nutrient absorption, all of which have a certain impact on the synthesis of coagulation factors in the body (42–44). The imbalance of tumor, coagulation, and inflammation in the bloodstream can promote tumor growth, invasion, and metastasis by causing coagulation disorders (45).

In summary, nomograms that include the five risk factors mentioned above demonstrate superior identification of early recurrence in patients with AFP-NHCC and elevation of DCP level. In addition, in patients with a high risk of early recurrence predicted by nomogram, repeat surgical resection, liver transplantation, and other adjunctive therapy options should be considered as an alternative treatment with shorter follow-up intervals.

Although this nomogram has good predictive performance, this study has some limitations. First of all, this study was a retrospective study conducted in a single center, and selection and indication bias are inevitable. Secondly, the establishment of our model is based on HCC patients receiving hepatectomy in the early stage, and whether it applies to middle to end-stage HCC patients remain to be verified. Thirdly, in this study, most patients had viral etiologies, but recent years have seen an increase in non-viral etiologies, which may lead to variations in tumor marker behavior. These variations could affect the generalizability of our findings, primarily applicable to patients with viral etiologies. Future studies should include a larger cohort of patients with non-viral etiologies and consider potential interactions between different etiologies to validate and extend our findings, enhancing their applicability and accuracy in a broader clinical context. Finally, our model lacks external validation. Future multi-center trials are still needed for further analysis.

## Conclusion

In summary, we established and validated a novel nomogram by multivariate Cox regression analysis to predict early recurrence in patients with AFP-NHCC and elevated DCP levels after resection. The nomogram, which includes five independent risk factors: age, tumor number, tumor size, γ-GT, and PT, demonstrates better predictive ability, and our predictive model is simple to calculate and contains readily available clinical indicators, which can greatly help physicians make personalized treatment decisions for this subgroup patient.

## Data availability statement

The raw data supporting the conclusions of this article will be made available by the authors, without undue reservation.

## Ethics statement

The studies involving humans were approved by Ethics Committee of Beijing You’an Hospital, Capital Medical University. The studies were conducted in accordance with the local legislation and institutional requirements. As a low-risk study by the Helsinki protocol, written informed consent was waived by the same local committee.

## Author contributions

JL: Formal analysis, Methodology, Writing – original draft. QW: Data curation, Writing – original draft. YY: Writing – review & editing. LS: Writing – original draft. GZ: Methodology, Writing –review & editing. GL: Conceptualization, Writing – original draft, Writing – review & editing. RJ: Conceptualization, Supervision, Writing – original draft, Writing – review & editing.
